# Lipid Bodies as Sites of Prostaglandin E2 Synthesis During Chagas Disease: Impact in the Parasite Escape Mechanism

**DOI:** 10.3389/fmicb.2018.00499

**Published:** 2018-03-20

**Authors:** Patrícia E. de Almeida, Daniel A. M. Toledo, Gabriel S. C. Rodrigues, Heloisa D’Avila

**Affiliations:** ^1^Laboratory of Cellular Biology, Department of Biology, Federal University of Juiz de Fora, Juiz de Fora, Brazil; ^2^Minas Gerais Federal Institute, Belo Horizonte, Brazil

**Keywords:** *T. cruzi*, lipid droplets, prostaglandin, infectious diseases, inflammation, lipid mediators, parasite replication, Chagas disease

## Abstract

During Chagas disease, the *Trypanosoma cruzi* can induce some changes in the host cells in order to escape or manipulate the host immune response. The modulation of the lipid metabolism in the host phagocytes or in the parasite itself is one feature that has been observed. The goal of this mini review is to discuss the mechanisms that regulate intracellular lipid body (LB) biogenesis in the course of this parasite infection and their meaning to the pathophysiology of the disease. The interaction host–parasite induces LB (or lipid droplet) formation in a Toll-like receptor 2-dependent mechanism in macrophages and is enhanced by apoptotic cell uptake. Simultaneously, there is a lipid accumulation in the parasite due to the incorporation of host fatty acids. The increase in the LB accumulation during infection is correlated with an increase in the synthesis of PGE_2_ within the host cells and the parasite LBs. Moreover, the treatment with fatty acid synthase inhibitor C75 or non-steroidal anti-inflammatory drugs such as NS-398 and aspirin inhibited the LB biogenesis and also induced the down modulation of the eicosanoid production and the parasite replication. These findings show that LBs are organelles up modulated during the course of infection. Furthermore, the biogenesis of the LB is involved in the lipid mediator generation by both the macrophages and the parasite triggering escape mechanisms.

## Introduction

Chagas disease represents an infectious condition classified by the World Health Organization (WHO) as a neglected illness. It is caused by the protozoan *Trypanosoma cruzi* and presents several symptoms, leading to a continuous inflammatory process that results in the replacement of functional health tissues by connective tissue, and thereafter, function loss of tissues and organs, which may lead to death (Teixeira et al., 1978, 2002; Parada et al., 1997; Rodriguez-Salas et al., 1998; Huang et al., 1999; Machado et al., 2008).

**FIGURE 1:**
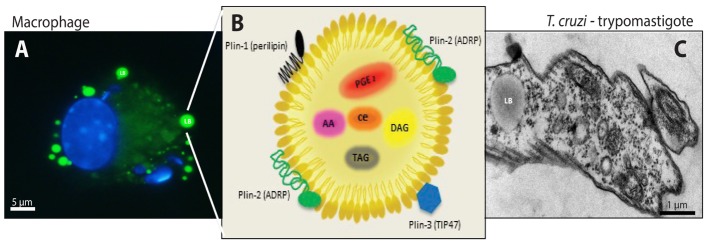
Lipid bodies (LBs) biogenesis and components in both the host cell cytoplasm during the interaction and/or infection with *T. cruzi* and in the trypomastigotes forms of *T. cruzi*. **(A)** LBs accumulation (green) in murine infected macrophage after staining with BODIPY^®^ 493/503. Nuclei of macrophage and internalized parasites were stained with DAPI (4′,6-diamidino-2-phenylindole; blue). **(B)** Schematic representation of the structural composition of a LB. Colored objects represent LBs surface-bound proteins located in the phospholipid monolayer. Prostaglandin E2 (PGE) 2, arachidonic acid (AA), diacilclycerols (DAG), triacylglycerols (TAG), and cholesterol esters (CE) are found in the neutral lipid core. **(C)** Electron micrograph showing a LB in the trypomastigote form of *T. cruzi*. From: Melo, RCN (courtesy); Toledo, DAM and D’Avila, H.

Studies in *T. cruzi* experimental infection models have established a strong immunological response in the acute phase, characterized by an intense infiltration of activated macrophages with the ability to process and present antigens, cytokines synthesis, and give co-stimulatory signals demonstrating their essential function in innate immune responses, in order to control the parasite multiplication and elimination (Teixeira et al., 2002). A distinguishing aspect of Chagas disease-triggered macrophages is the increased numbers of distinct cytoplasmic organelles called lipid bodies (LBs) (**Figure 1**) (Melo et al., 2003; D’Avila et al., 2011).

Lipid bodies are lipid rich-organelles that have been found in almost all organisms from bacteria to humans (Alvarez et al., 1996; Waltermann et al., 2005; Murphy, 2012). In mammalians, LBs are found in the major part of leukocytes and other cellular types, such as endothelial cells, fibroblasts, and mastocytes (Dvorak et al., 1993; Bozza et al., 2007) and can be involved directly or indirectly in numerous cellular functions, such as lipid metabolism, membrane traffic, intracellular signaling and the production of several inflammatory mediators (Bozza et al., 2007). LBs within infected cells are involved in the production of inflammatory mediators which can potentially inhibit the host Th1 response, thus, modulating parasite growth (Snijdewint et al., 1993; Kalinski, 2012). Interestingly, a recent study established that *T. cruzi* LBs are also active and producing immunosuppressive inflammatory mediators which may represent not only an evasion strategy but also a survival factor exhibited by the parasite (Toledo et al., 2016).

The purpose of this mini review is to present the recent progress in elucidating the structure, formation mechanisms and functions of intracellular LBs within both infected host cells and the protozoan parasite *T. cruzi*, as well as their impact on the host response and parasite escape mechanism during Chagas disease.

## Lipid Body Characterization and Structure

Lipid bodies, also known as lipid droplets or adiposomes, are multi-functional organelles associated with lipid homeostasis in virtually all cells (**Figure 1**). Although, the cellular and molecular mechanisms of LBs biogenesis remain to be determined; it is currently known that the endoplasmic reticulum (ER) structure may have an important role during LB biogenesis. In eukaryotic cells, LBs are formed *de novo* from the ER (Jacquier et al., 2011; Kassan et al., 2013; Choudhary et al., 2015). The most accepted model suggests that it was as a *building model*, where enzymes, such as diacyltransferase DGAT1 and DGAT2, produce triacylglycerols (TAG). Moreover, these enzymes are involved in lipid metabolism localized in specific compartments of the ER, favoring the synthesis of neutral lipid between the two membrane leaflets of the ER, producing a hydrophobic neutral lipid core (Murphy and Vance, 1999; Bozza et al., 2009; Walther et al., 2017). After reaching a determined size, nascent LBs carried with proteins lacking trans-membrane spanning domains bud off from ER into the cytoplasm and finally the lipids are coated by a phospholipid monolayer from the cytoplasmic leaflet of the ER membrane (Murphy, 1999, 2001; Martin and Parton, 2005; Bozza et al., 2009; Walther et al., 2017).

In general, the LB structure consists of a neutral lipid core, containing TAG and cholesterol ester (CE) in its majority, surrounded by an outer monolayer of phospholipids because LBs besides being heterogeneous organelles also lack a true delimiting unit membrane structure (Tauchi-Sato et al., 2002). Moreover, LBs are structured by perilipin (PLIN) family proteins, including perilipin/PLIN1, PLIN2/ADRP (adipose differentiation-related protein), PLIN3/TIP47 (tail-interacting protein of 47 KDa) (**Figure 1B**) (Brasaemle et al., 1997; Wolins et al., 2006; Dalen et al., 2007; Welte, 2007). The protein content can be diverse once proteomic studies have shown ribosomal, mitochondrial, and vesicular transport proteins, such as Ras-associated binding protein (RAB)s, ADP-ribosylation factor (ARF)s, caveolins and ER components compartmentalized in the LBs, suggesting their role in fusion and fission with other LBs or organelles, as well as cell signaling and inflammatory mediator proteins under different conditions. However, the lipid and protein content depend on the cell type and condition of the cellular activation (Dvorak et al., 1993; Bozza et al., 1997; Yu et al., 1998, 2000; Wu et al., 2000; Fujimoto et al., 2001; Chen et al., 2002; Umlauf et al., 2004; Ozeki et al., 2005; Bartz et al., 2007; Bostrom et al., 2007; Hodges and Wu, 2010).

## Lipid Body Formation During *T. cruzi* Infection

The mechanism of formation of LBs in host cells is a highly regulated event. Upon leukocytes activation LBs are formed rapidly in response to different stimuli and pathological conditions, such as infection by distinct pathogens: mycobacteria (Cardona and Ausina, 2000; D’Avila et al., 2006; Almeida et al., 2009, 2014; Mattos et al., 2010, 2011), virus (Ferguson et al., 2017) or protozoan, such as *T. cruzi* (Melo et al., 2003; D’Avila et al., 2011), *Leishmania major* (Rabhi et al., 2016), *L. amazonensis* (Pinheiro et al., 2009; Lecoeur et al., 2013), and *Toxoplasma gondii* (Gomes et al., 2014; Mota et al., 2014).

During *in vivo* studies in *T. cruzi* infection, it was demonstrated that this disease promotes an important inflammatory response featured by intense macrophage migration to the infectious sites, mainly the heart (Melo and Machado, 2001; Melo et al., 2003). Melo et al. (2006) showed LBs enhancement in inflammatory macrophages associated with increased myocardial parasitism (Melo et al., 2006). Moreover, during *T. cruzi* infection, LBs show a diversity electron-density, which suggest a diverse composition associated with recruitment and/or *in situ* production of lipid inflammatory mediators (Melo et al., 2003, 2006).

In macrophages, the *T. cruzi* internalization potentiates LB biogenesis; however, the phagocytosis is neither sufficient nor essential for triggering the biogenesis. It has been demonstrated that after a 24 h period of infection with *T. cruzi*, peritoneal macrophages with internalized parasites, as well as non-parasitized cells show increased number of LBs compared to control (D’Avila et al., 2011). Although not fully elucidated, the formation of LBs in host macrophages seems to involve the pathogen recognition by surface receptors, as well as paracrine signaling that soluble factors secreted by parasites or infected cells might induce LB biogenesis in non-parasitized cells.

**FIGURE 2:**
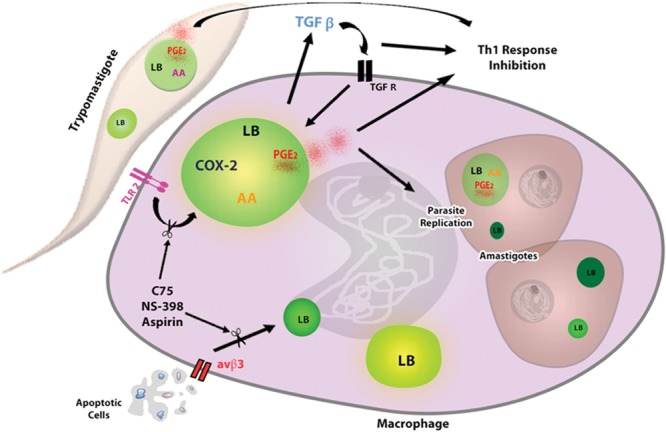
Lipid bodies formation in response to interaction macrophage- *T. cruzi* favors parasite replication. The uptake of trypomastigotes through TLR2 induces LBs formation in macrophages, which is potentiated by phagocytosis of apoptotic cells through αvβ3 receptor. The interaction of parasite–macrophage also induces LBs accumulation in extracellular trypomastigotes and intracellular amastigotes, which can serve as lipid sources for parasite growth. In addition, the TGF-β produced by infected macrophages acts autocrinally contributing for LBs increase. New formed LBs from parasite and macrophage are sites for PGE_2_ synthesis, because they compartmentalize the substrate (AA) and the enzymes as (COX-2 and PGE_2_ synthase) for their production. PGE_2_ is a potent lipid mediator that, together with TGF- β, potentially reduces the host Th1 immune response, thus decreasing the microbicidal capacity of the macrophage. The macrophages treatment with Aspirin, NS-398 or C75 can inhibit LBs accumulation and LBs-derived PGE_2_ synthesis, controlling the parasite replication. AA, arachidonic acid; COX-2, cyclooxygenase -2, TGF-βR, TGF- βR receptor.

Our group demonstrated that, in murine macrophages, the *in vitro T. cruzi* infection induced LBs formation through recognition via toll like receptor 2 (TLR-2) (**Figure 2**) (D’Avila et al., 2011). In fact, some groups of researchers have identified different molecular motifs from this parasite able of activating TLRs in macrophages, such as Glycosylphosphatidylinositol-anchored mucin-like glycoproteins (tGPI-mucin) present in the parasite membrane and capable of inducing the inflammatory response through an activation of TLR2 (Almeida et al., 1999; Campos et al., 2001; Gravina et al., 2013). However, the identification of downstream signaling pathways involved in this processes during *T. cruzi* infection needs to be more elucidated. TLR4 has also been involved in the immune response during the first stage of infection (Rodrigues et al., 2012); nonetheless, it was not able to mediate the LB formation in macrophages (D’Avila et al., 2011).

During *T. cruzi* infection, the induction of apoptosis, especially of T and B lymphocytes (Freire-de-Lima et al., 2000; DosReis, 2011) and neutrophils (Magalhaes et al., 2017) represents an important mechanism that contributes to the parasite replication, due to the immunomodulatory effects on the host immune response (Decote-Ricardo et al., 2017). Consequently, the efferocytosis or phagocytic clearance of these apoptotic cells by macrophages has profound consequences on innate and adaptive immune responses in inflamed tissues (Elliott et al., 2017). Moreover, it has been shown that the formation of LBs during *T. cruzi* infection in macrophages is potentiated in the presence of apoptotic, but not necrotic or living cells (D’Avila et al., 2011).

The uptake of apoptotic cells through the α_v_β_3_ integrin (vitronectin receptor) is critical in the induction of LBs during *T. cruzi* infection (**Figure 2**). In addition, the treatment with flavoridin, a desintegrin that blocks binding via a_v_β_3_, completely abolished the LB-formation induced by the apoptotic cells uptake (D’Avila et al., 2011). Furthermore, some groups have shown that the interaction of apoptotic cells and phagocytic cells induces the production of cytokines such as IL-10 and TGF-β (Voll et al., 1997; Xiao et al., 2006, 2008) causing these cells to be more permissive to *T. cruzi* infection (Freire-de-Lima et al., 2000; D’Avila et al., 2011). Studies *in vitro* have shown that the TGF- β produced by macrophages could induce LBs in these cells. The use anti-TGF β1 neutralizing antibody inhibited the secretion of TGF- β, and abolished the LB formation induced by this cytokine, demonstrating that this mediator can directly trigger LB formation (**Figure 2**) (D’Avila et al., 2011). Even though the attachment of other co-receptors cannot be ruled out, these data suggest that efferocytosis by macrophages through α_v_β_3_ receptor triggers TGF-β1-dependent potentiating the LB biogenesis.

## Lipid Body Formation in the Protozoan *T. cruzi*

In recent years, it has become of interest the study of the biogenesis, structure, composition, and function of LBs formed within protists parasites, such as *T. cruzi*. These parasites are able to acquire host lipids or to codify their own lipid biosynthesis machinery, thus allowing LBs biogenesis independently of their host (D’Avila et al., 2012; Herker and Ott, 2012).

Toledo et al. (2016) showed that metacyclic trypomastigote forms from *T. cruzi*, co-cultured with peritoneal macrophages for 1 h had enhanced LB biogenesis, suggesting that the interaction of infective forms of parasite with inflammatory host leukocytes such as macrophages might quickly modulate the LB formation in the *T. cruzi* (**Figure 2**). Moreover, ultrastructural analyses of LBs from amastigote forms inside macrophages, showed the presence of a typical monolayer of phospholipids with varied electron-density, similar for the one of the mammals cells. In addition, the electron density was dependent on the cell activation state and the LBs from the amastigotes inside heart macrophages, during *in vivo* infection, were more electron-dense, than the LBs from peritoneal macrophages, during *in vitro* infection (Toledo et al., 2016).

Furthermore, it has been showed that the arachidonic acid (AA) is a potent inductor of LB formation in eukaryotic cells (Weller et al., 1991b; Bozza et al., 1996) and that these organelles incorporate AA, mostly esterified in phospholipids (Weller and Dvorak, 1985; Weller et al., 1991a). Interestingly, trypomastigotes forms of *T. cruzi* stimulated by AA *in vitro* presented an enhanced number of LBs when compared to unstimulated parasites in a time- and dose-dependent manner, with a peak at 24 h of *in vitro* stimulation. Raman spectroscopy and MALDI-TOF mass spectroscopy confirmed that both parasites stimulated by AA can incorporate a higher content of unsaturated fatty acids, such as AA inside parasite LBs (Toledo et al., 2016). These organelles, formed as the outcome of host interaction, suggest that the high content of AA can be captured from host cell by the parasite (**Figure 2**).

## Lipid Bodies Are Specialized in the Eicosanoids Synthesis in Both Parasite and Host Cells

As described before, LBs can accumulate AA, suggesting that these LBs are potentially efficient to initiate intracellular signaling pathways that culminate in the formation of lipid inflammatory mediators, such as eicosanoids (Weller and Dvorak, 1985; Weller et al., 1991a). Prostaglandins (PG) are eicosanoids derived from AA, which are converted by cyclooxygenase (COX-1 and COX-2) into PGH_2_, which in turn is converted *in vivo* and *in vitro* into various arachidonate metabolites, such as PGD_2_, PGE_2_, and PGF_2a_ (Hayaishi and Urade, 2002; Miller, 2006). The PGE_2_ sustains homeostatic functions and mediates pathogenic mechanisms, including the inflammatory response associated with parasitic disease (Kubata et al., 2007). In fact, previous works documented LBs as sites of compartmentalization of eicosanoid-forming enzymes (Yu et al., 1998; Bozza et al., 2002; D’Avila et al., 2006, 2011), and *in situ* production of eicosanoids, such as leukotrienes and prostaglandins, were really identified in these organelles within activated cells during an inflammatory situation (Bandeira-Melo et al., 2001; Pacheco et al., 2002; Vieira-de-Abreu et al., 2005; D’Avila et al., 2006).

Earlier works have demonstrated that macrophages infected by *T. cruzi* were positively immunostained for COX-2, and COX-2 expression was increased when macrophages were co-cultured with apoptotic cells (Freire-de-Lima et al., 2000; D’Avila et al., 2011). In addition, D’Avila et al. (2011) confirmed that COX-2 is localized within LBs as well as in the perinuclear membrane in infected cells. Using Eicosacell technique, a strategy developed for direct *in situ* immunolocalization of eicosanoid synthesis (Bandeira-Melo et al., 2011), new formed PGE_2_ was produced in LBs induced by *T. cruzi* infection in the presence of apoptotic cells (D’Avila et al., 2011).

After the findings on the synthesis of PGE_2_ in LB-induced by *T. cruzi* in macrophages, it was showed that LBs from trypomatigotes forms of *T. cruzi*, are capable to incorporate AA and might be sources of PGE_2_ synthesis, suggesting an activation of the AA cascade and a likely pathway for PGE_2_ production in the parasite (Toledo et al., 2016). Moreover, the parasites produce PGs, like eukaryotic cells possessing the enzymatic machineries for PG biosynthesis (Daugschies and Joachim, 2000; Kubata et al., 2002; Noverr et al., 2003). However, the homologs of mammalian COX have not been found in any parasitic protozoan so far, although proteins called COX-like enzymes, that are similar to the mammalian COX-1 and COX-2 have already been identified (Kubata et al., 2002). Indeed, trypomastigotes forms of *T. cruzi*, stimulated by AA led to quantitative increases in LBs biogenesis in parallel with PGE_2_ secretion and PGE_2_ synthase expression (Toledo et al., 2016). Thus, the co-localization of LB and PGE_2_ sites within stimulated trypomastigotes, give credence to the LBs as organelles to the sites for newly formed PGE_2_ during the activation (Toledo et al., 2016). This is also true for the *T. cruzi* infection in macrophages (D’Avila et al., 2011). These data suggest that LBs may be the source of lipid and inflammatory mediators, in response to the host–parasite interaction. Furthermore, PGE_2_ may be a powerful immunomodulator and acts in the immunosuppression that occurs during *T. cruzi* infection, indicating a function for PGs from *T*. *cruzi* in the Chagas disease pathogenesis.

## Lipid Body Inhibition as Infection Control Strategy

Based on the effects that *T. cruzi* infection and apoptotic cell uptake cause on LBs formation in the host cell, it has been investigated whether modulation of the formation of this organelle could impact the replication of the parasite (D’Avila et al., 2011). It was tested the effect of two non-steroidal anti-inflammatory drugs (NSAIDs), aspirin (COX-1 and COX-2 inhibitor) and NS- 398 (COX-2 inhibitor) which, in addition to their COX inhibitory effect, also inhibit COX-independent LB formation (Bozza et al., 1996, 2002). Both aspirin and NS-398 inhibited the LB biogenesis in infected macrophages in the presence or absence of apoptotic cells, suppressing the *T. cruzi* effects on LB-derived PGE_2_ synthesis, and reversing the enhancement on parasite replication induced by apoptotic cells (**Figure 2**). Therefore, the biogenesis of the LBs in both the *T. cruzi* infection and in the parasite interaction has a direct role in the ability of the macrophages to synthesize increased amounts of PGE_2_, which may have an impact on the course of the disease (D’Avila et al., 2011).

In parallel, LB biogenesis seems to request *de novo* lipid synthesis in a cellular mechanism controlled by fatty acid synthase (Schmid et al., 2005; D’Avila et al., 2006; Accioly et al., 2008). Therefore, the fatty acid synthase inhibitor C75 significantly inhibited LB biogenesis induced by *T. cruzi* infection, with or without the uptake of apoptotic cells, through a mechanism independent of the inhibition of the COX-2 enzyme (**Figure 2**). Remarkably, it was demonstrated that the treatment with C75 also reversed the parasite replication in macrophages as well as the formation of LBs (D’Avila et al., 2011).

In conclusion, it is safe to say that these organelles show an important role in the inflammatory response, especially against intracellular pathogens, since their biogenesis leads to the production of inflammatory mediators, suppressing the macrophage effectiveness to respond and reduce its capacity to eliminate the parasite and control the infection. In this mini review, we analyzed the structure, composition and function of the LBs in the parasite and host cell during *T. cruzi* infection (Melo et al., 2003; D’Avila et al., 2011). The increases in LB numbers in *T. cruzi*, associated with changes in LB ultrastructure highlight the fact that LBs parasites are also plastic, dynamic and active organelles, which are efficient in modifying their structure and composition in line with immune cell activation mechanisms.

## Concluding Remarks

Studies have investigated the intriguing formation of LBs, both in the host cell and in the parasite itself (D’Avila et al., 2011; Toledo et al., 2016). Newly formed host LBs are distinguished for their efficiency to synthesize lipid inflammatory mediators, such as PGE_2_ and to compartmentalize eicosanoid-forming enzymes, such as COX-2 (Yu et al., 1998; Bozza et al., 2002; D’Avila et al., 2006, 2011).

Host leukocytes LBs triggered by *T. cruzi* infection and increased by the phagocytosis of apoptotic cells are accepted not only as inflammatory organelles and structural markers of parasite-induced cell activation, but also as organelles efficient in the orchestration of the host cell metabolism (D’Avila et al., 2011). A recent work supports the idea that the *T. cruzi* itself is capable of producing LB-derived PGE_2_ after contact with the host cell to facilitate its own survival (Toledo et al., 2016). This is evidence that parasites have adapted to their lipid hosts modulation mechanisms by taking advantage of the cellular metabolism favoring the diseases progression. However, the effects of modulating the formation of LBs by distinct drugs and their influence in the control of parasite replication experimentally, suggest mechanisms that could help in the discovery of new effective therapies for Chagas disease.

## Author Contributions

PA and HD drafted and edited the manuscript. DMT edited the figures. PA, DMT, GR, and HD wrote and approved the final version of the paper.

## Conflict of Interest Statement

The authors declare that the research was conducted in the absence of any commercial or financial relationships that could be construed as a potential conflict of interest.

## References

[B1] Accioly M. T. Pacheco P. Maya-Monteiro C. M. Carrossini N. Robbs B. K. Oliveira S. S. (2008). Lipid bodies are reservoirs of cyclooxygenase-2 and sites of prostaglandin-E2 synthesis in colon cancer cells. *Cancer Res.* 68 1732–1740. 10.1158/0008-5472.CAN-07-1999 18339853

[B2] Almeida I. C. Gazzinelli R. Ferguson M. A. Travassos L. R. (1999). Trypanosoma cruzi mucins: potential functions of a complex structure. *Mem. Inst. Oswaldo Cruz* 94(Suppl. 1) 173–176. 10.1590/S0074-02761999000700023 10677709

[B3] Almeida P. E. Roque N. R. Magalhaes K. G. Mattos K. A. Teixeira L. Maya-Monteiro C. (2014). Differential TLR2 downstream signaling regulates lipid metabolism and cytokine production triggered by *Mycobacterium bovis* BCG infection. *Biochim. Biophys. Acta* 1841 97–107. 10.1016/j.bbalip.2013.10.008 24120921

[B4] Almeida P. E. Silva A. R. Maya-Monteiro C. M. Torocsik D. D’Avila H. Dezso B. (2009). *Mycobacterium bovis* bacillus Calmette-Guerin infection induces TLR2-dependent peroxisome proliferator-activated receptor gamma expression and activation: functions in inflammation, lipid metabolism, and pathogenesis. *J. Immunol.* 183 1337–1345. 10.4049/jimmunol.0900365 19561094

[B5] Alvarez H. M. Mayer F. Fabritius D. Steinbuchel A. (1996). Formation of intracytoplasmic lipid inclusions by *Rhodococcus opacus* strain PD630. *Arch. Microbiol.* 165 377–386. 10.1007/s002030050341 8661931

[B6] Bandeira-Melo C. Phoofolo M. Weller P. F. (2001). Extranuclear lipid bodies, elicited by CCR3-mediated signaling pathways, are the sites of chemokine-enhanced leukotriene C4 production in eosinophils and basophils. *J. Biol. Chem.* 276 22779–22787. 10.1074/jbc.M101436200 11274187

[B7] Bandeira-Melo C. Weller P. F. Bozza P. T. (2011). EicosaCell - an immunofluorescent-based assay to localize newly synthesized eicosanoid lipid mediators at intracellular sites. *Methods Mol. Biol.* 689 163–181. 10.1007/978-1-60761-950-5_10 21153792 PMC3679533

[B8] Bartz R. Zehmer J. K. Zhu M. Chen Y. Serrero G. Zhao Y. (2007). Dynamic activity of lipid droplets: protein phosphorylation and GTP-mediated protein translocation. *J. Proteome Res.* 6 3256–3265. 10.1021/pr070158j 17608402

[B9] Bostrom P. Andersson L. Rutberg M. Perman J. Lidberg U. Johansson B. R. (2007). SNARE proteins mediate fusion between cytosolic lipid droplets and are implicated in insulin sensitivity. *Nat. Cell Biol.* 9 1286–1293. 10.1038/ncb1648 17922004

[B10] Bozza P. T. Magalhaes K. G. Weller P. F. (2009). Leukocyte lipid bodies - Biogenesis and functions in inflammation. *Biochim. Biophys. Acta* 1791 540–551. 10.1016/j.bbalip.2009.01.005 19416659 PMC2693476

[B11] Bozza P. T. Melo R. C. Bandeira-Melo C. (2007). Leukocyte lipid bodies regulation and function: contribution to allergy and host defense. *Pharmacol. Ther.* 113 30–49. 10.1016/j.pharmthera.2006.06.006 16945418

[B12] Bozza P. T. Pacheco P. Yu W. Weller P. F. (2002). NS-398: cyclooxygenase-2 independent inhibition of leukocyte priming for lipid body formation and enhanced leukotriene generation. *Prostaglandins Leukot. Essent. Fatty Acids* 67 237–244. 10.1054/plef.2002.0425 12401438

[B13] Bozza P. T. Payne J. L. Morham S. G. Langenbach R. Smithies O. Weller P. F. (1996). Leukocyte lipid body formation and eicosanoid generation: cyclooxygenase-independent inhibition by aspirin. *Proc. Natl. Acad. Sci. U.S.A.* 93 11091–11096. 10.1073/pnas.93.20.11091 8855314 PMC38289

[B14] Bozza P. T. Yu W. Weller P. F. (1997). Mechanisms of formation and function of eosinophil lipid bodies: inducible intracellular sites involved in arachidonic acid metabolism. *Mem. Inst. Oswaldo Cruz* 92(Suppl. 2) 135–140. 10.1590/S0074-02761997000800018 9698925

[B15] Brasaemle D. L. Barber T. Wolins N. E. Serrero G. Blanchette-Mackie E. J. Londos C. (1997). Adipose differentiation-related protein is an ubiquitously expressed lipid storage droplet-associated protein. *J. Lipid Res.* 38 2249–2263. 9392423

[B16] Campos M. A. Almeida I. C. Takeuchi O. Akira S. Valente E. P. Procopio D. O. (2001). Activation of toll-like receptor-2 by glycosylphosphatidylinositol anchors from a protozoan parasite. *J. Immunol.* 167 416–423. 10.4049/jimmunol.167.1.416 11418678

[B17] Cardona P. J. Ausina V. (2000). Histopathology of tuberculosis. Approximation to the clinical course of lung lesions in animal experimentation models induced with aerosols. *Arch Bronconeumol.* 36 645–650. 10.1016/S0300-2896(15)30087-9 11171437

[B18] Chen J. S. Greenberg A. S. Wang S. M. (2002). Oleic acid-induced PKC isozyme translocation in RAW 264.7 macrophages. *J. Cell. Biochem.* 86 784–791. 10.1002/jcb.10266 12210744

[B19] Choudhary V. Ojha N. Golden A. Prinz W. A. (2015). A conserved family of proteins facilitates nascent lipid droplet budding from the ER. *J. Cell Biol.* 211 261–271. 10.1083/jcb.201505067 26504167 PMC4621845

[B20] D’Avila H. Freire-de-Lima C. G. Roque N. R. Teixeira L. Barja-Fidalgo C. Silva A. R. (2011). Host cell lipid bodies triggered by *Trypanosoma cruzi* infection and enhanced by the uptake of apoptotic cells are associated with prostaglandin E(2) generation and increased parasite growth. *J. Infect. Dis.* 204 951–961. 10.1093/infdis/jir432 21849292

[B21] D’Avila H. Melo R. C. Parreira G. G. Werneck-Barroso E. Castro Faria Neto H. C. Bozza P. T. (2006). *Mycobacterium bovis* BCG induces TLR 2-mediated formation of lipid bodies: intracellular domains for eicosanoid synthesis in vivo. *J. Immunol.* 176 3087–3097. 10.4049/jimmunol.176.5.3087 16493068

[B22] D’Avila H. Toledo D. A. Melo R. C. (2012). Lipid bodies: inflammatory organelles implicated in host-*Trypanosoma cruzi* interplay during innate immune responses. *Mediators Inflamm.* 2012:478601. 10.1155/2012/478601 22619483 PMC3350868

[B23] Dalen K. T. Dahl T. Holter E. Arntsen B. Londos C. Sztalryd C. (2007). LSDP5 is a PAT protein specifically expressed in fatty acid oxidizing tissues. *Biochim. Biophys. Acta* 1771 210–227. 10.1016/j.bbalip.2006.11.011 17234449

[B24] Daugschies A. Joachim A. (2000). Eicosanoids in parasites and parasitic infections. *Adv. Parasitol.* 46 181–240. 10.1016/S0065-308X(00)46009-4 10761556

[B25] Decote-Ricardo D. Nunes M. P. Morrot A. Freire-de-Lima C. G. (2017). Implication of apoptosis for the pathogenesis of *Trypanosoma cruzi* infection. *Front. Immunol.* 8:518. 10.3389/fimmu.2017.00518 28536576 PMC5422484

[B26] DosReis G. A. (2011). Evasion of immune responses by *Trypanosoma cruzi*, the etiological agent of Chagas disease. *Braz. J. Med. Biol. Res.* 44 84–90. 10.1590/S0100-879X2011007500005 21243314

[B27] Dvorak A. M. Weller P. F. Harvey V. S. Morgan E. S. Dvorak H. F. (1993). Ultrastructural localization of prostaglandin endoperoxide synthase (cyclooxygenase) to isolated, purified fractions of guinea pig peritoneal macrophage and line 10 hepatocarcinoma cell lipid bodies. *Int. Arch. Allergy Immunol.* 101 136–142. 10.1159/000236511 8508051

[B28] Elliott M. R. Koster K. M. Murphy P. S. (2017). Efferocytosis signaling in the regulation of macrophage inflammatory responses. *J. Immunol.* 198 1387–1394. 10.4049/jimmunol.1601520 28167649 PMC5301545

[B29] Ferguson D. Zhang J. Davis M. A. Helsley R. N. Vedin L. L. Lee R. G. (2017). The lipid droplet-associated protein perilipin 3 facilitates hepatitis C virus-driven hepatic steatosis. *J. Lipid Res.* 58 420–432. 10.1194/jlr.M073734 27941027 PMC5282958

[B30] Freire-de-Lima C. G. Nascimento D. O. Soares M. B. P. Bozza P. T. Castro-Faria-Neto H. C. De Mello F. G. (2000). Uptake of apoptotic cells drives the growth of a pathogenic trypanosome in macrophages. *Nature* 403 199–203. 10.1038/35003208 10646605

[B31] Fujimoto T. Kogo H. Ishiguro K. Tauchi K. Nomura R. (2001). Caveolin-2 is targeted to lipid droplets, a new ”membrane domain” in the cell. *J. Cell Biol.* 152 1079–1085. 10.1083/jcb.152.5.1079 11238462 PMC2198803

[B32] Gomes A. F. Magalhaes K. G. Rodrigues R. M. de Carvalho L. Molinaro R. Bozza P. T. (2014). Toxoplasma gondii-skeletal muscle cells interaction increases lipid droplet biogenesis and positively modulates the production of IL-12, IFN-g and PGE2. *Parasit. Vectors* 7:47. 10.1186/1756-3305-7-47 24457118 PMC3904159

[B33] Gravina H. D. Antonelli L. Gazzinelli R. T. Ropert C. (2013). Differential use of TLR2 and TLR9 in the regulation of immune responses during the infection with *Trypanosoma cruzi*. *PLoS One* 8:e63100. 10.1371/journal.pone.0063100 23650544 PMC3641106

[B34] Hayaishi O. Urade Y. (2002). Prostaglandin D2 in sleep-wake regulation: recent progress and perspectives. *Neuroscientist* 8 12–15. 10.1177/107385840200800105 11843094

[B35] Herker E. Ott M. (2012). Emerging role of lipid droplets in host/pathogen interactions. *J. Biol. Chem.* 287 2280–2287. 10.1074/jbc.R111.300202 22090026 PMC3268388

[B36] Hodges B. D. Wu C. C. (2010). Proteomic insights into an expanded cellular role for cytoplasmic lipid droplets. *J. Lipid Res.* 51 262–273. 10.1194/jlr.R003582 19965608 PMC2803228

[B37] Huang H. Chan J. Wittner M. Jelicks L. A. Morris S. A. Factor S. M. (1999). Expression of cardiac cytokines and inducible form of nitric oxide synthase (NOS2) in *Trypanosoma cruzi*-infected mice. *J. Mol. Cell. Cardiol.* 31 75–88. 10.1006/jmcc.1998.0848 10072717

[B38] Jacquier N. Choudhary V. Mari M. Toulmay A. Reggiori F. Schneiter R. (2011). Lipid droplets are functionally connected to the endoplasmic reticulum in *Saccharomyces cerevisiae*. *J. Cell Sci.* 124(Pt 14) 2424–2437. 10.1242/jcs.076836 21693588

[B39] Kalinski P. (2012). Regulation of immune responses by prostaglandin E2. *J. Immunol.* 188 21–28. 10.4049/jimmunol.1101029 22187483 PMC3249979

[B40] Kassan A. Herms A. Fernandez-Vidal A. Bosch M. Schieber N. L. Reddy B. J. (2013). Acyl-CoA synthetase 3 promotes lipid droplet biogenesis in ER microdomains. *J. Cell Biol.* 203 985–1001. 10.1083/jcb.201305142 24368806 PMC3871434

[B41] Kubata B. K. Duszenko M. Martin K. S. Urade Y. (2007). Molecular basis for prostaglandin production in hosts and parasites. *Trends Parasitol.* 23 325–331. 10.1016/j.pt.2007.05.005 17531535

[B42] Kubata B. K. Kabututu Z. Nozaki T. Munday C. J. Fukuzumi S. Ohkubo K. (2002). A key role for old yellow enzyme in the metabolism of drugs by *Trypanosoma cruzi*. *J. Exp. Med.* 196 1241–1251. 10.1084/jem.20020885 12417633 PMC2194105

[B43] Lecoeur H. Giraud E. Prevost M. C. Milon G. Lang T. (2013). Reprogramming neutral lipid metabolism in mouse dendritic leucocytes hosting live *Leishmania amazonensis* amastigotes. *PLoS Negl. Trop. Dis.* 7:e2276. 10.1371/journal.pntd.0002276 23785538 PMC3681733

[B44] Machado F. S. Souto J. T. Rossi M. A. Esper L. Tanowitz H. B. Aliberti J. (2008). Nitric oxide synthase-2 modulates chemokine production by *Trypanosoma cruzi*-infected cardiac myocytes. *Microbes Infect.* 10 1558–1566. 10.1016/j.micinf.2008.09.009 18951994 PMC2643379

[B45] Magalhaes L. M. D. Viana A. de Jesus A. C. Chiari E. Galvao L. Gomes J. A. (2017). Distinct *Trypanosoma cruzi* isolates induce activation and apoptosis of human neutrophils. *PLoS One* 12:e0188083. 10.1371/journal.pone.0188083 29176759 PMC5703490

[B46] Martin S. Parton R. G. (2005). Caveolin, cholesterol, and lipid bodies. *Semin. Cell Dev. Biol.* 16 163–174. 10.1016/j.semcdb.2005.01.007 15797827

[B47] Mattos K. A. D’Avila H. Rodrigues L. S. Oliveira V. G. Sarno E. N. Atella G. C. (2010). Lipid droplet formation in leprosy: toll-like receptor-regulated organelles involved in eicosanoid formation and *Mycobacterium leprae* pathogenesis. *J. Leukoc. Biol.* 87 371–384. 10.1189/jlb.0609433 19952355

[B48] Mattos K. A. Lara F. A. Oliveira V. G. Rodrigues L. S. D’Avila H. Melo R. C. (2011). Modulation of lipid droplets by *Mycobacterium leprae* in Schwann cells: a putative mechanism for host lipid acquisition and bacterial survival in phagosomes. *Cell. Microbiol.* 13 259–273. 10.1111/j.1462-5822.2010.01533.x 20955239

[B49] Melo R. C. D’Avila H. Fabrino D. L. Almeida P. E. Bozza P. T. (2003). Macrophage lipid body induction by Chagas disease in vivo: putative intracellular domains for eicosanoid formation during infection. *Tissue Cell* 35 59–67. 10.1016/S0040-8166(02)00105-2 12589730

[B50] Melo R. C. Fabrino D. L. Dias F. F. Parreira G. G. (2006). Lipid bodies: structural markers of inflammatory macrophages in innate immunity. *Inflamm. Res.* 55 342–348. 10.1007/s00011-006-5205-0 16977381

[B51] Melo R. C. Machado C. R. (2001). *Trypanosoma cruzi*: peripheral blood monocytes and heart macrophages in the resistance to acute experimental infection in rats. *Exp. Parasitol.* 97 15–23. 10.1006/expr.2000.4576 11207110

[B52] Miller S. B. (2006). Prostaglandins in health and disease: an overview. *Semin. Arthritis Rheum.* 36 37–49. 10.1016/j.semarthrit.2006.03.005 16887467

[B53] Mota L. A. Roberto Neto J. Monteiro V. G. Lobato C. S. Oliveira M. A. Cunha M. (2014). Culture of mouse peritoneal macrophages with mouse serum induces lipid bodies that associate with the parasitophorous vacuole and decrease their microbicidal capacity against *Toxoplasma gondii*. *Mem. Inst. Oswaldo Cruz* 109 767–774. 10.1590/0074-0276140119 25317704 PMC4238769

[B54] Murphy D. J. (1999). Production of novel oils in plants. *Curr. Opin. Biotechnol.* 10 175–180. 10.1016/S0958-1669(99)80031-7 10209131

[B55] Murphy D. J. (2001). The biogenesis and functions of lipid bodies in animals, plants and microorganisms. *Prog. Lipid Res.* 40 325–438. 10.1016/S0163-7827(01)00013-3 11470496

[B56] Murphy D. J. (2012). The dynamic roles of intracellular lipid droplets: from archaea to mammals. *Protoplasma* 249 541–585. 10.1007/s00709-011-0329-7 22002710

[B57] Murphy D. J. Vance J. (1999). Mechanisms of lipid-body formation. *Trends Biochem. Sci.* 24 109–115. 10.1016/S0968-0004(98)01349-8 10203758

[B58] Noverr M. C. Erb-Downward J. R. Huffnagle G. B. (2003). Production of eicosanoids and other oxylipins by pathogenic eukaryotic microbes. *Clin. Microbiol. Rev.* 16 517–533. 10.1128/CMR.16.3.517-533.2003 12857780 PMC164223

[B59] Ozeki S. Cheng J. Tauchi-Sato K. Hatano N. Taniguchi H. Fujimoto T. (2005). Rab18 localizes to lipid droplets and induces their close apposition to the endoplasmic reticulum-derived membrane. *J. Cell Sci.* 118(Pt 12) 2601–2611. 10.1242/jcs.02401 15914536

[B60] Pacheco P. Bozza F. A. Gomes R. N. Bozza M. Weller P. F. Castro-Faria-Neto H. C. (2002). Lipopolysaccharide-induced leukocyte lipid body formation in vivo: innate immunity elicited intracellular loci involved in eicosanoid metabolism. *J. Immunol.* 169 6498–6506. 10.4049/jimmunol.169.11.6498 12444160

[B61] Parada H. Carrasco H. A. Anez N. Fuenmayor C. Inglessis I. (1997). Cardiac involvement is a constant finding in acute Chagas’ disease: a clinical, parasitological and histopathological study. *Int. J. Cardiol.* 60 49–54. 10.1016/S0167-5273(97)02952-5 9209939

[B62] Pinheiro R. O. Nunes M. P. Pinheiro C. S. D’Avila H. Bozza P. T. Takiya C. M. (2009). Induction of autophagy correlates with increased parasite load of *Leishmania amazonensis* in BALB/c but not C57BL/6 macrophages. *Microbes Infect.* 11 181–190. 10.1016/j.micinf.2008.11.006 19070676

[B63] Rabhi S. Rabhi I. Trentin B. Piquemal D. Regnault B. Goyard S. (2016). Lipid droplet formation, their localization and dynamics during *Leishmania major* macrophage infection. *PLoS One* 11:e0148640. 10.1371/journal.pone.0148640 26871576 PMC4752496

[B64] Rodrigues M. M. Oliveira A. C. Bellio M. (2012). The immune response to *Trypanosoma cruzi*: role of toll-like receptors and perspectives for vaccine development. *J. Parasitol. Res.* 2012:507874. 10.1155/2012/507874 22496959 PMC3306967

[B65] Rodriguez-Salas L. A. Klein E. Acquatella H. Catalioti F. Davalos V. V. Gomez-Mancebo J. R. (1998). Echocardiographic and clinical predictors of mortality in chronic Chagas’ Disease. *Echocardiography* 15 271–278. 10.1111/j.1540-8175.1998.tb00607.x 11175040

[B66] Schmid B. Rippmann J. F. Tadayyon M. Hamilton B. S. (2005). Inhibition of fatty acid synthase prevents preadipocyte differentiation. *Biochem. Biophys. Res. Commun.* 328 1073–1082. 10.1016/j.bbrc.2005.01.067 15707987

[B67] Snijdewint F. G. Kalinski P. Wierenga E. A. Bos J. D. Kapsenberg M. L. (1993). Prostaglandin E2 differentially modulates cytokine secretion profiles of human T helper lymphocytes. *J. Immunol.* 150 5321–5329. 8390534

[B68] Tauchi-Sato K. Ozeki S. Houjou T. Taguchi R. Fujimoto T. (2002). The surface of lipid droplets is a phospholipid monolayer with a unique Fatty acid composition. *J. Biol. Chem.* 277 44507–44512. 10.1074/jbc.M207712200 12221100

[B69] Teixeira A. R. Teixeira G. Macedo V. Prata A. (1978). *Trypanosoma cruzi*-sensitized T-lymphocyte mediated 51CR release from human heart cells in Chagas’ disease. *Am. J. Trop. Med. Hyg.* 27 1097–1107. 10.4269/ajtmh.1978.27.1097 83110

[B70] Teixeira M. M. Gazzinelli R. T. Silva J. S. (2002). Chemokines, inflammation and *Trypanosoma cruzi* infection. *Trends Parasitol.* 18 262–265. 10.1016/S1471-4922(02)02283-3 12036740

[B71] Toledo D. A. Roque N. R. Teixeira L. Milan-Garces E. A. Carneiro A. B. Almeida M. R. (2016). Lipid body organelles within the parasite *Trypanosoma cruzi*: a role for intracellular arachidonic acid metabolism. *PLoS One* 11:e0160433. 10.1371/journal.pone.0160433 27490663 PMC4973985

[B72] Umlauf E. Csaszar E. Moertelmaier M. Schuetz G. J. Parton R. G. Prohaska R. (2004). Association of stomatin with lipid bodies. *J. Biol. Chem.* 279 23699–23709. 10.1074/jbc.M310546200 15024010

[B73] Vieira-de-Abreu A. Assis E. F. Gomes G. S. Castro-Faria-Neto H. C. Weller P. F. Bandeira-Melo C. (2005). Allergic challenge-elicited lipid bodies compartmentalize in vivo leukotriene c4 synthesis within eosinophils. *Am. J. Respir. Cell Mol. Biol.* 33 254–261. 10.1165/rcmb.2005-0145OC 15947420 PMC2715315

[B74] Voll R. E. Herrmann M. Roth E. A. Stach C. Kalden J. R. Girkontaite I. (1997). Immunosuppressive effects of apoptotic cells. *Nature* 390 350–351. 10.1038/37022 9389474

[B75] Waltermann M. Hinz A. Robenek H. Troyer D. Reichelt R. Malkus U. (2005). Mechanism of lipid-body formation in prokaryotes: how bacteria fatten up. *Mol. Microbiol.* 55 750–763. 10.1111/j.1365-2958.2004.04441.x 15661001

[B76] Walther T. C. Chung J. Farese R. V. Jr. (2017). Lipid droplet biogenesis. *Annu. Rev. Cell Dev. Biol.* 33 491–510. 10.1146/annurev-cellbio-100616-060608 28793795 PMC6986389

[B77] Weller P. F. Dvorak A. M. (1985). Arachidonic acid incorporation by cytoplasmic lipid bodies of human eosinophils. *Blood* 65 1269–1274. 3922452

[B78] Weller P. F. Monahan-Earley R. A. Dvorak H. F. Dvorak A. M. (1991a). Cytoplasmic lipid bodies of human eosinophils: subcelular isolation and analysis of arachidonic incorporation. *Am. J. Pathol.* 138 141–148. 1846262 PMC1886053

[B79] Weller P. F. Ryeom S. W. Picard S. T. Ackerman S. J. Dvorak A. M. (1991b). Cytoplasmic lipid bodies of neutrophils: formation induced by cis-unsaturated fatty acids and mediated by protein kinase C. *J. Cell Biol.* 113 137–146. 10.1083/jcb.113.1.137 1901065 PMC2288908

[B80] Welte M. A. (2007). Proteins under new management: lipid droplets deliver. *Trends Cell Biol.* 17 363–369. 10.1016/j.tcb.2007.06.004 17766117

[B81] Wolins N. E. Brasaemle D. L. Bickel P. E. (2006). A proposed model of fat packaging by exchangeable lipid droplet proteins. *FEBS Lett.* 580 5484–5491. 10.1016/j.febslet.2006.08.040 16962104

[B82] Wu C. C. Howell K. E. Neville M. C. Yates J. R. III. McManaman J. L. (2000). Proteomics reveal a link between the endoplasmic reticulum and lipid secretory mechanisms in mammary epithelial cells. *Electrophoresis* 21 3470–3482. 10.1002/1522-2683(20001001)21:16<3470::AID-ELPS3470>3.0.CO;2-G 11079566

[B83] Xiao Y. Q. Freire-de-Lima C. G. Janssen W. J. Morimoto K. Lyu D. Bratton D. L. (2006). Oxidants selectively reverse TGF-beta suppression of proinflammatory mediator production. *J. Immunol.* 176 1209–1217. 10.4049/jimmunol.176.2.1209 16394011

[B84] Xiao Y. Q. Freire-de-Lima C. G. Schiemann W. P. Bratton D. L. Vandivier R. W. Henson P. M. (2008). Transcriptional and translational regulation of TGF-beta production in response to apoptotic cells. *J. Immunol.* 181 3575–3585. 10.4049/jimmunol.181.5.3575 18714031 PMC2583327

[B85] Yu W. Bozza P. T. Tzizik D. M. Gray J. P. Cassara J. Dvorak A. M. (1998). Co-compartmentalization of MAP kinases and cytosolic phospholipase A2 at cytoplasmic arachidonate-rich lipid bodies. *Am. J. Pathol.* 152 759–769. 9502418 PMC1858398

[B86] Yu W. Cassara J. Weller P. F. (2000). Phosphatidylinositide 3-kinase localizes to cytoplasmic lipid bodies in human polymorphonuclear leukocytes and other myeloid-derived cells. *Blood* 95 1078–1085. 10648425

