# Corrigendum: Lipid Bodies as Sites of Prostaglandin E2 Synthesis During Chagas Disease: Impact in the Parasite Escape Mechanism

**DOI:** 10.3389/fmicb.2018.01168

**Published:** 2018-05-31

**Authors:** Patrícia E. de Almeida, Daniel A. M. Toledo, Gabriel S. C. Rodrigues, Heloisa D'Avila

**Affiliations:** ^1^Laboratory of Cellular Biology, Department of Biology, Federal University of Juiz de Fora, Juiz de Fora, Brazil; ^2^Minas Gerais Federal Institute, Belo Horizonte, Brazil

**Keywords:** *T. cruzi*, lipid droplets, prostaglandin, infectious diseases, inflammation, lipid mediators, parasite replication, Chagas disease

In our mini review article there was an error in the infected macrophage included in Figure 1A and the name of one of the authors of the Figure 1C was omitted.

**Figure 1:**
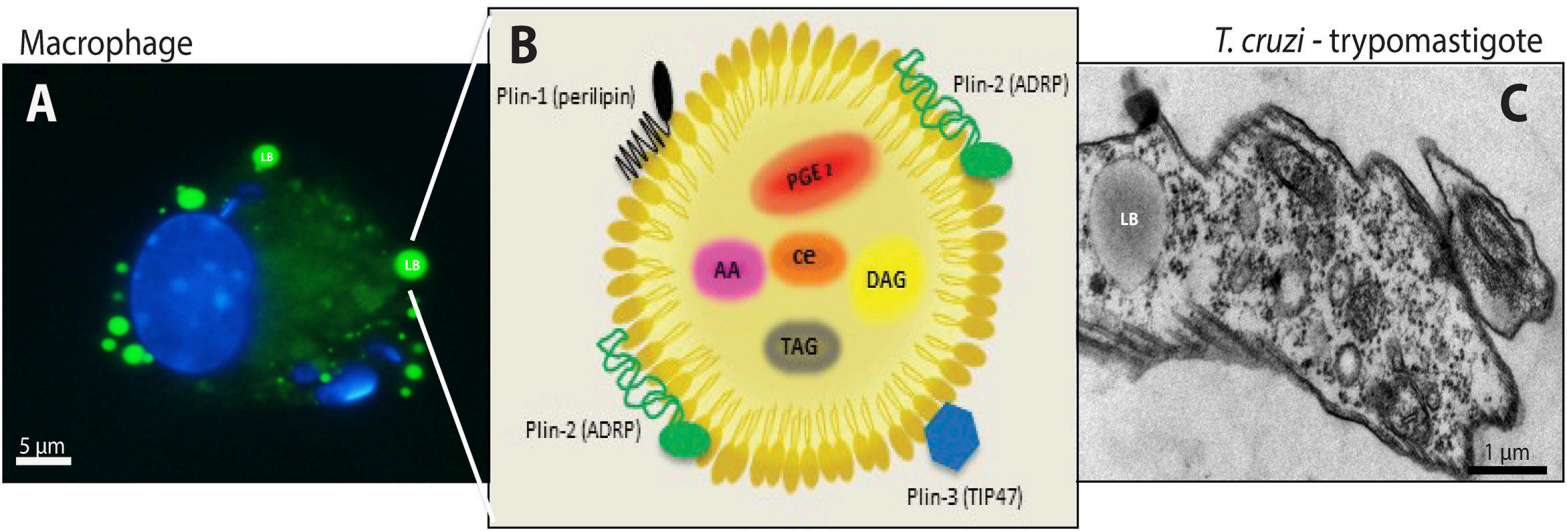
Lipid bodies (LBs) biogenesis and components in both the host cell cytoplasm during the interaction and/or infection with *T. cruzi* and in the trypomastigotes forms of *T. cruzi*. **(A)** LBs accumulation (green) in murine infected macrophage after staining with BODIPY® 493/503. Nuclei of macrophage and internalized parasites were stained with DAPI (4′,6- diamidino-2-phenylindole; blue). **(B)** Schematic representation of the structural composition of a LB. Colored objects represent LBs surface-bound proteins located in the phospholipid monolayer. Prostaglandin E_2_ (PGE) _2_, Arachidonic acid (AA), Diacilclycerols (DAG), Triacylglycerols (TAG) and cholesterol esters (CE) are found in the neutral lipid core. **(C)** Electron micrograph showing a LB in the trypomastigote form of *T. cruzi*. From: Melo, RCN (courtesy); Toledo, DAM and D'Avila, H.

The correct version of Figure 1A, its legend, and the name of the authors of Figure 1C appear below. The authors apologize for the mistake. This error does not change the scientific conclusions of the mini review article in any way.

The original article has been updated.

## Conflict of interest statement

The authors declare that the research was conducted in the absence of any commercial or financial relationships that could be construed as a potential conflict of interest.

